# The Antitumor Potential of λ-Carrageenan Oligosaccharides on Gastric Carcinoma by Immunomodulation

**DOI:** 10.3390/nu15092044

**Published:** 2023-04-24

**Authors:** Min Tang, Leilei Zhai, Juanjuan Chen, Feng Wang, Haimin Chen, Wei Wu

**Affiliations:** 1 State Key Laboratory for Managing Biotic and Chemical Threats to the Quality and Safety of Agro-Products, Ningbo University, Ningbo 315211, Chinachenhaimin@nbu.edu.cn (H.C.); 2 Collaborative Innovation Center for Zhejiang Marine High-Efficiency and Healthy Aquaculture, Ningbo University, Ningbo 315211, China; 3 Department of Laboratory Medicine, Ningbo Medical Center Lihuili Hospital, Ningbo University, Ningbo 315040, China

**Keywords:** λ-carrageenan oligosaccharides, gastric carcinoma, Par-4 signaling pathway, immunoregulation

## Abstract

Gastric carcinoma is a frequently detected malignancy worldwide, while its mainstream drugs usually result in some adverse reactions, including immunosuppression. λ-carrageenan oligosaccharides (COS) have attracted increasing attention as potential anticancer agents due to their ability to enhance immune function. Our current work assessed the antitumor mechanism of λ-COS using BGC-823 cells. Our findings indicated that λ-COS alone did not have a significant impact on BGC-823 cells in vitro; however, it was effective in inhibiting tumor growth in vivo. When THP-1 cells were pre-incubated with λ-COS and used to condition the medium, BGC-823 cells in vitro displayed a concentration-dependent induction of cell apoptosis, nuclear damage, and the collapse of mitochondrial transmembrane potential. These findings suggested that the antineoplastic effect of λ-COS was primarily due to its immunoenhancement property. Treatment with λ-COS was found to significantly enhance the phagocytic capability of macrophages, increase the secretion of TNF-α and IFN-γ, and improve the indexes of spleen and thymus in BALB/c mice. In addition, λ-COS was found to inhibit the growth of BGC-823-derived tumors in vitro by activating the Par-4 signaling pathway, which may be stimulated by the combination of TNF-α and IFN-γ. When used in combination with 5-FU, λ-COS demonstrated enhanced anti-gastric carcinoma activity and improved the immunosuppression induced by 5-FU alone. These findings suggested that λ-COS could be used as an immune-modulating agent for chemotherapy.

## 1. Introduction

Gastric carcinoma is characterized by high mortality and has been ranked the fifth most frequently diagnosed cancer worldwide [1]. Chemotherapy remains one of the most efficacious therapeutic regimens for various tumors [2]. Anticancer drugs, such as 5-fluorouracil (5-FU), cisplatin, adriamycin, and mitomycin, play a vital role in chemotherapy. Among these drugs, 5-FU is the standard chemotherapy regimen against gastric carcinoma [3]. 5-FU is an analog of uracil in which a fluorine atom replaces the hydrogen atom at the C-5 position. This modification can affect the activity of thymidylate synthase and inhibit DNA synthesis, leading to cell apoptosis induction [4]. However, the side effects of 5-FU are far from negligible. It is frequently reported that oral mucositis and diarrhea are two main side effects induced by 5-FU [5]. Additionally, 5-FU can cause immunosuppression by impairing the immune system’s functions in the body. Current research has suggested that repeated cycles of 5-FU treatment can damage the functions of cytotoxic T-cells, which are unable to fully recover after chemotherapy suspension [6]. As a result, scientists are increasingly focusing on mitigating the side effects of 5-FU by exploring antitumor agents with lower toxicity that may produce synergistic treatment effects that enhance the curative efficacy of 5-FU treatment on gastric carcinoma.

**Figure 1:**
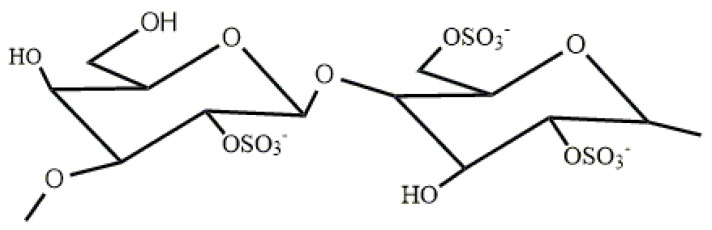
The repeating disaccharide unit of λ-CGN.

Carrageenan (CGN) represents a variety of sulfated polysaccharides extracted from marine red algae (Rhodophyta). There are three primary types of CGN, namely lambda (λ)-, iota (ι)-, and kappa (κ)-CGN. λ-CGN is primarily composed of D-galactose units with alternating alpha-1,3 and beta-1,4-glucoside bonds and does not contain the 3,6-endoether-galactose unit (Figure 1) [7]. Among these polysaccharides, λ-CGN polysaccharides have the highest antitumor and immunostimulant activities. The tumor inhibition rate of low-molecular-weight λ-CGN is usually higher than high-molecular-weight λ-CGN in H22- or S180-bearing mice. The lowest molecular weight of λ-CGN bestows it with the highest tumor inhibition rate [8]. CGN has been considered a food additive in the food industry for a long time. However, the application of polysaccharides is greatly limited in medicine due to their considerable molecular weight, low absorption rate, metabolic difficulties, and other adverse factors. Upon hydrolysis of CGN to carrageenan oligosaccharides (COS), the molecular weight is reduced, water solubility is improved, and the active groups are more fully exposed. Moreover, COS has been found to exhibit lower toxicity and fewer side effects compared to CGN, showing enhanced physiological activity [9]. As a result, COS, and other oligosaccharides, have gained significant attention in the biomedical field. Hugo et al. demonstrated that λ-COS could inhibit the migration of MDA-MB-231 breast cancer cells [10]. Zeng et al. have proven that Celastrol-conjugated chitosan oligosaccharide can inhibit tumor growth, induce apoptosis, and effectively suppress tumor metastasis in human pancreatic cancer cells [11]. Bryan et al. have found that hyaluronan oligosaccharides can induce apoptosis in various types of cancer cells while leaving normal cells unaffected, even rendering chemo-resistant cells sensitive to drugs when co-treated with hyaluronan oligosaccharides [12]. Additionally, alginate oligosaccharides have been shown to attenuate the proliferation, migration, and invasion of human prostate cancer cells through the suppression of the Hippo/YAP/c-Jun pathway [13]. These oligosaccharides are extensively studied as adjuvant drugs for cancer treatment regimens on the international market. 

The antineoplastic activity of oligosaccharides is realized by direct tumor inhibition, synergistic chemotherapy, and immune improvement [14]. Among the commonly used immune cells, macrophages are intensively employed as the research model for the antitumor activity of oligosaccharides in vitro as activated macrophages can release tumor necrosis factor-α (TNF-α), a crucial cytotoxic mediator that can wreck tumor cells. Gustavo et al. have discovered that κ-, ι-, and λ-COS exhibit cytotoxic effects on LM2 tumor cells through the apoptotic pathway [15]. Furthermore, Tastio et al. have demonstrated that κ- and λ-COS induce apoptosis in adenocarcinomas, particularly showing high apoptosis induction in human colorectal adenocarcinoma RKO cells [16]. Despite these signs of progress, there are few relevant reports on the anti-neoplastic activity and mechanism of COS against gastric carcinoma.

Our previous study found that Caco-2 colon cancer cells are not sensitive to CGN [17]. In contrast, a very low concentration of CGN (≦10 μg/mL) can induce apoptosis and damage cell layer integrity in the co-culture model of Caco-2 and THP-1 cells via the Toll-like receptor 4 (TLR4) and ERK1/2-mediated promotion of TNF-α secretion [17,18]. This finding suggests that the antitumor activity of λ-COS is related to the immunoregulatory impacts on macrophages. However, IFN-γ is rarely reported in related research. In our current study, we assess the antitumor activity of λ-COS from an immunomodulatory perspective, using both in vitro and in vivo models. Our goal is to provide more robust evidence for the medical utilization of λ-COS and its potential adjunctive effect in inhibiting gastric carcinoma.

## 2. Materials and Methods 

### 2.1. Preparation and Purification of λ-COS

λ-COS with low (∼2 kDa) molecular weight was prepared from native λ-CGN extracted from *Kappaphycopsis cottonii* (formerly *Eucheuma cottonii*) (Rhodophyta) (purchased from Sigma-Aldrich, St. Louis, MO, USA). Native λ-CGN was dissolved in distilled water (1% *w*/*v*), and the mixture was vigorously stirred and heated to 65 °C. Next, 0.05 mol/L of concentrated hydrochloric acid was mixed with the λ-CGN solution and incubated at 90 °C for 30 min. After neutralization with NaOH, a hollow fiber cartridge with an MW cut-off of 10 kDa (Sartorius Inc., Vivaflow, Germany) was adopted to filter the solution. The filtrate was subjected to a second ultrafiltration (MW cut-off of 5 kDa). The preparation of λ-COS was precipitated with four volumes of anhydrous ethanol and dried at −60 °C using a vacuum freeze dryer. The mean molecular weight of λ-COS was examined employing high-performance gel permeation chromatography with an Agilent Technologies 1260 series HPLC machine (Agilent Technologies, Santa Clara, CA, USA). The chromatographic column used was Tskgel G3000PWxl gel column, with a sample concentration of 5 mg/mL and a sample size of 20 μL. The detector used was a differential refraction detector at a temperature of 35 °C. For MS analysis, we used an electrospray ionization source with a spray voltage of 2.8 kV, an ion transfer capillary temperature of 320 °C, trocar umbilicus balloon (N2) pressure of 25 psi, and Aux gas pressure of 5 arbitrary units. Positive ion mode detection was used with Level 1 Full Scan mode, which scanned the range from 300 to 3000 *m*/*z*. The gelatin-barium chloride method was performed to determine the sulfate content [19]. Dissolved in deionized water to final concentrations of 0.5–2 mg/mL, λ-COS was scanned using a Varioskan Flash (Thermo Fisher Scientific, Waltham, MA, USA) within the range of 200 to 500 nm to test their purity.

### 2.2. Cell Lines and Reagents

The human gastric adenocarcinoma cells BGC-823 and human myeloid leukemia mononuclear cells THP-1 cells were purchased from China Center for Type Culture Collection (CTCC, Wuhan, China). Cells were cultured in Roswell Park Memorial Institute-1640 medium (RPMI-1640, Gibco BRL, Grand Island, NY, USA) containing 10% fetal bovine serum (FBS, Gibco BRL, Grand Island, NY, USA) in a humidified atmosphere (37 °C, 5% CO_2_). In addition, phorbol-12-myristate-13-acetate (PMA) was purchased from Solarbio (Peking, China). 

### 2.3. Collection of Conditioned Medium

λ-COS was dissolved in RPMI-1640 medium at indicated concentrations (1, 2, 5, 10, and 20 μg/mL) and subjected to sterilization using a 0.22-μm membrane filter. THP-1 cells were cultivated in 6-well plates (1 × 10^6^ cells per well). After exposure to 0.1 µg/mL PMA for 72 h, THP-1 cells would transdifferentiate into macrophage-like cells. Then the THP-1-derived macrophages were rinsed with PBS and incubated with indicated concentrations of λ-COS for 48 h. Finally, the supernatants were harvested as a conditioned medium, centrifugated at 3000× *g* for 10 min at 4 °C, and stored at −20 °C for later use [20]. In addition, the medium without λ-COS was used to cultivate the cells of the control group.

### 2.4. Cell Counting Kit-8 (CCK-8) Assay

To evaluate the effects of λ-COS and conditioned medium on the growth of cancer cells, eight different cell lines, such as liver cancer HepG2 cells, colon cancer epithelial Caco-2 cells and HT-29 cells, small glioma BV-2 cells, breast cancer MCF-7 cells, lung cancer PC-9 cells, THP-1 cells, and BGC-823 cells, were cultivated in 96-well plates (1 × 10^4^ cells/well). 5-FU solution (dissolved in RMPI-1640 complete medium, 10 μg/mL) and RPMI-1640 complete medium were considered positive and negative controls, respectively, followed by overnight incubation. Subsequently, the supernatant was discarded, and various concentrations of λ-COS (dissolved in RMPI-1640 complete medium, 1, 2, 5, 10, and 20 μg/mL) or conditioned medium solutions were added, followed by incubation for 24 h. Then, the culture medium was aspirated and 20 μL of CCK-8 reagent and 180 μL RPMI-1640 medium were added to each well, followed by 2 h of incubation. Finally, the absorbance of the samples was detected by Varioskan Flash at 450 nm (Thermo Fisher Scientific, Waltham, MA, USA). Triple experiments were performed and the obtained data were normalized to the control group during analysis.

### 2.5. DAPI Staining

BGC-823 cells (4 × 10^4^ cells/well) were cultivated in 24-well plates for 12 h. Then, indicated conditioned medium or 10 μg/mL 5-FU solution was added. After 24 h of incubation, the cells were rinsed with PBS and then were fixed with 4% paraformaldehyde solution at room temperature for a half hour. Next, the cells were rinsed with PBS again and exposed to 10 μg/mL DAPI (Beyotime, Shanghai, China) at 37 °C for 20 min in the dark. The images of stained cells were captured by a fluorescence microscope (Olympus Optical Co., Ltd., Tokyo, Japan).

### 2.6. Annexin-V/PI Apoptosis Detection

Annexin-V/PI staining was performed to evaluate cell apoptotic rates. The BGC-823 cells were cultured in a 6-well plate (1 × 10^6^ cells/well) and exposed to indicated conditioned medium or 10 μg/mL 5-FU for 24 h. Subsequently, the cells were resuspended in the Annexin-V-FITC and PI binding buffer (Beyotime, Shanghai, China) for a quarter of an hour with no light exposure. Thus, the stained cells could be detected by Beckman Gallios Flow Cytometer (Beckman Coulter Inc., Brea, CA, USA). In addition, 488 nm was the excitation wavelength of FITC and PI, while the emission wavelengths of FITC and PI were 530 nm and 630 nm, respectively. 

### 2.7. Measurement of Mitochondrial Transmembrane Potential

The mitochondrial transmembrane potential was detected by adopting 5,5′,6,6′-tetrachloro-1,1′,3,3′-tetraethylbenzimidazolcarbocyanine iodide (JC-1) staining. Briefly, the cells treated as indicated were incubated with a staining solution (Beyotime, Shanghai, China) containing JC-1 probe at 37 °C for 20 min in the dark. The mixture was then rinsed twice with JC-1 staining buffer (Beyotime, Shanghai, China). Images were observed under the fluorescence microscope (Olympus Optical Co., Ltd., Tokyo, Japan). 

### 2.8. Enzyme-Linked Immunosorbent Assay (ELISA) 

The conditioned medium was harvested after the 48 h incubation with λ-COS. Two-site sandwich ELISA was performed to quantify TNF-α and IFN-γ in the conditioned medium according to the manufacturer’s instructions (Multisciences, Hangzhou, China). 

### 2.9. Neutral Red Uptake Assay

A neutral red uptake assay was used to determine the phagocytic ability of THP-1-derived macrophages. Briefly, THP-1 cells were seeded in 96-well plates at a density of 1 × 10^4^ cells per well and treated with 0.1 μg/mL PMA for 72 h, followed by the incubation with the indicated concentrations of λ-COS (1, 2, 5, 10, and 20 μg/mL) or 5-FU (10 μg/mL) for another 48 h, respectively. After discarding the culture supernatant, the cells would be incubated with 0.1% neutral red (dissolved in PBS) for 1 h at 37 °C. Then the cells were rinsed with warm PBS three times to dislodge dissociative neutral red and lysed overnight in a buffer consisting of ethanol and glacial acetic acid at the ratio of 1:1 at 4 °C in the dark. Finally, the spectrophotometric absorbance of each group was detected at a wavelength of 540 nm. The data were expressed as the percentage of the control group.

### 2.10. Western Blotting Analysis

The cells treated as indicated were harvested, lysed in radioimmunoprecipitation assay (RIPA) buffer, and centrifuged at 12,000× *g* for 15 min at 4 °C to collect the proteins in the cell. The nuclear fraction was isolated employing the NE-PER nuclear and cytoplasmic extraction kit (Rockford, IL, USA). Equal amounts of proteins were separated using sodium dodecyl sulfate-polyacrylamide gel electrophoresis (SDS-PAGE, 15% gels) and electro-transferred onto polyvinylidene fluoride membranes. The membranes were exposed to primary antibodies against cleaved-caspase-8 (1:1000, Cell Signaling Technology Inc., Danvers, MA, USA), Par-4 (1:1000, Abcam Inc., Cambridge, MA, USA), NF-κB/p65 (1:1000, Abcam Inc., Cambridge, MA, USA), Akt (1:1000, Cell Signaling Technology Inc., Danvers, MA, USA), p-Akt (Ser473, 1:1000, Cell Signaling Technology Inc., Danvers, MA, USA), Bcl-2 (1:1000, Santa Cruz Biotechnology Inc., Santa Cruz, CA, USA), GADPH (1:1000, Santa Cruz Biotechnology Inc., CA, USA), and Histone (1:2000, Cell Signaling Technology Inc., Danvers, MA, USA) at 4 °C overnight, followed by a 1 h incubation with appropriate horseradish peroxidase (HRP)-conjugated secondary antibodies (goat anti-rabbit IgG, 1:5000, goat anti-mouse IgG, 1:8000, Santa Cruz Biotechnology Inc., CA, USA) at room temperature. Besides, the membranes were rinsed with TBST five times. The probed proteins were visualized by Western Bright ECL-HRP Substrate (Advansta Inc., Menlo Park, CA, USA).

### 2.11. Xenografted Model and Treatment Procedures

A total of 80 female BALB/c mice (4–6 weeks old, 15–18 g) were obtained from Beijing Weitong Lihua Experimental Animal Technology Co., Ltd. (Peking, China). Animals living in a temperature- and humidity-controlled environment (21 ± 2 °C and approximately 60%, respectively) were housed in the Animal Center of Ningbo University Medicine College (Ningbo, China) under a half-day light/half-day dark photoperiod (lights on at 08:00 AM) with four mice in each cage. Moreover, mice were given free access to water and standard chow. To generate the xenograft animal models, about 2 × 10^7^ BGC-823 cells in 100 μL PBS were subcutaneously injected into the left front axilla of BALB/c mice (the normal group was excluded.). The mice were randomly assigned into seven groups, including the normal group, vehicle group, λ-COS groups (100 and 200 mg/kg), 5-FU group (20 mg/kg), and combined groups (100, 200 mg/kg λ-COS + 20 mg/kg 5-FU) with six mice in each group (after 3 days of tumor cell xenograft, the tumors were macroscopic in the subcutaneous area. Some mice that did not have visible tumors on day 3 were excluded from the experiment. So eventually, only 42 mice were used in the study, with six mice in each group). Moreover, 100 μL saline was injected into the peritoneum for the normal group and vehicle group. The λ-COS groups received intraperitoneal injections of 100 μL λ-COS at 100 mg/kg and 200 mg/kg. Since day 1, the intraperitoneal injection was given daily, and the tumor volume of mice was measured every 3 d. The volume of tumors was calculated using the formula as follows: tumor volume (mm^3^) = length (mm) × width (mm^2^)/2. After 3 weeks, the mice were anesthetized by isopentane and sacrificed by cervical dislocation after collecting blood samples from their eyeballs. The collected blood was placed in a centrifuge tube without anticoagulation and centrifuged at 2000–2500 rpm for 1–2 min, and the upper serum was collected. The tumor, thymus, and spleen were immediately harvested and weighed. The tumor inhibition rate was calculated with the formula (1 − average tumor weight in treated groups/average tumor weight in the vehicle group) × 100%. The immune indices, namely the thymus index and spleen index, were calculated using the following formula: thymus index = (the weight of thymus/the weight of mice); spleen index = (the weight of spleen/the weight of mice). The tumor samples were stored at −80 °C or preserved in 4% paraformaldehyde for further analysis. Parts of the liver sections were stained using a CGN-specific Alcian blue staining technique [21].

### 2.12. Histopathological Assessment

Briefly, 10% formalin was used to fix the tumor specimens for 24 h. Thus, the specimen could be embedded in paraffin. Sectioned into 4 µm slices, the tissue samples were stained by hematoxylin and eosin (H&E). The stained samples were observed by an experienced pathologist using the double-blind method under an optical microscope (Olympus Optical, Tokyo, Japan) to assess changes in the infiltration of inflammatory cells and necrotic areas of tumors.

### 2.13. In Vivo Phagocytosis Assay

Briefly, 4% mercaptoethyl starch broth solution was injected intraperitoneally into every BALB/c mouse in each group (*n* = 6). After 3 days, the peritoneal macrophages were harvested, and a neutral red uptake assay was used to evaluate their phagocytic ability. The data were expressed as the percentage of the normal group. 

### 2.14. Analysis of Cytokine Levels

Serum samples were obtained after the last administration of λ-COS and preserved at −80 °C before further analysis. Two-site sandwich ELISA was performed to quantify TNF-α and IFN-γ in the conditioned medium according to the manufacturer’s instructions (Multisciences, Hangzhou, China). 

### 2.15. Immunohistochemical Detection

The tumor tissue slices (4 µm) were de-paraffinized, hydrated, and incubated with 3% H_2_O_2_ for 10 min in the dark to block the endogenous peroxidase activity. Then the slices were soaked in 0.01 M citrate buffer (pH 6.0) for antigen retrieval and further probed overnight with the primary antibodies against Bcl-2 (1:1000, Santa Cruz Biotechnology Inc., CA, USA) and cleaved-caspase-8 (1:1000, Cell Signaling Technology Inc., Danvers, MA, USA) at 4 °C. Next, the slices were washed with PBS and then incubated with specific secondary antibodies for 1 h. Then the immunoreactive cells in the samples were stained with freshly prepared diaminobenzidine, followed by observation using an optical microscope after dehydration. 

### 2.16. Statistical Analysis

All data were analyzed with GraphPad Prism 8.0 software (GraphPad Prism, Inc., San Diego, CA, USA) and represented as mean ± standard deviation. All the results were compared using one-way ANOVA, and multiple comparisons were corrected by the Student–Newman–Keuls test. *p* < 0.05 was considered statistically significant. *p* < 0.01 was considered statistically highly significant.

## 3. Results

### 3.1. The Effects of λ-COS and Conditioned Medium on the Growth of BGC-823 Cells

**Figure 2:**
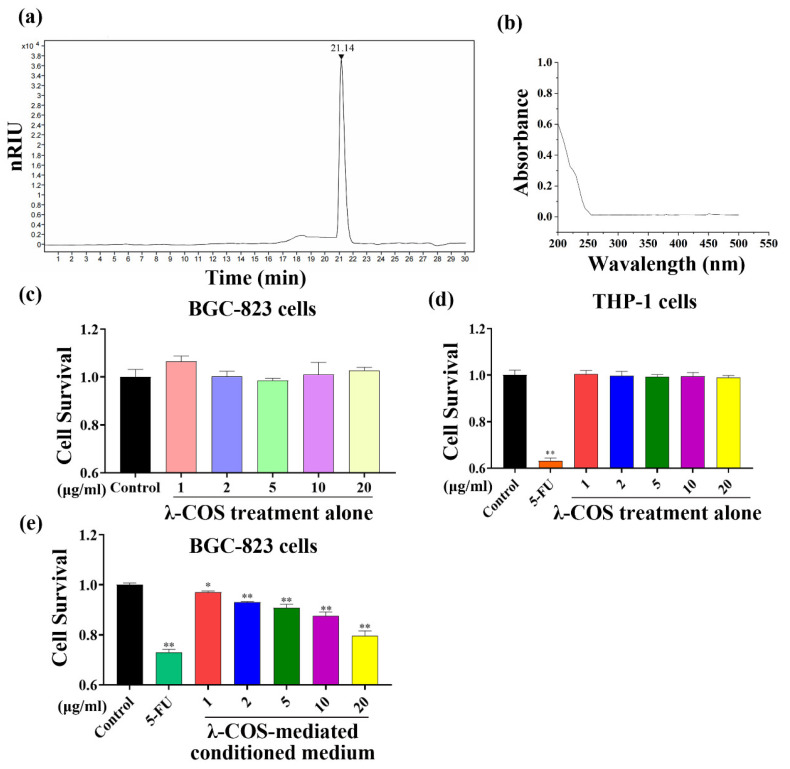
Characterization and effect of λ-COS on the proliferation of BGC-823 cells. (**a**) The HPLC chromatogram of λ-COS. (**b**) UV absorption spectra of λ-COS. (**c**) The survival rate of BGC-823 cells cultured with different concentrations of λ-COS was assessed by CCK-8 assay after 24 h incubation (*n* = 3). (**d**) The survival rate of THP-1 cells cultured with different concentrations of λ-COS was assessed by CCK-8 assay after 24 h of incubation (*n* = 3). (**e**) The survival rate of BGC-823 cells cultured with different concentrations of λ-COS-mediated conditioned medium was assessed via CCK-8 assay after 24 h incubation (*n* = 3). * *p* < 0.05 and ** *p* < 0.01 versus the control group.

λ-COS used for gavage in the study had an average molecular weight of 1.8 kDa with a sulfate content of 38.15%. The oligosaccharide structure information of 2~15 sugars in λ-COS was obtained by ESI-QqQ-MS (Figure 2a, Supplementary Table S1 and Supplementary Figures S1–S3). Besides, λ-COS showed no absorption at 260 nm or 280 nm, suggesting the nonexistence of nucleic acids and proteins in the samples (Figure 2b). Following incubation with λ-COS for 24 h, the CCK-8 assay indicated that the cell proliferation of eight different cell strains, namely liver cancer HepG2 cells, colon cancer epithelial Caco-2 cells and HT-29 cells, small glioma BV-2 cells, breast cancer MCF-7 cells, lung cancer PC-9 cells, monocytic lymphoma cells THP-1 cells, and gastric carcinoma BGC-823 cells, was not sensitive to λ-COS at the used concentrations in comparison with the control group (Figure 2c,d, Supplementary Figure S4). However, the growth of BGC-823 cells was inhibited by the conditioned medium mediated by various concentrations of λ-COS (1, 2, 5, 10, and 20 μg/mL) after incubation for 24 h. The highest inhibition rate of conditioned medium mediated by 20 μg/mL λ-COS reached 20.5 ± 2.1% compared with the control group (*p* < 0.01). In contrast, the cytotoxicity of conditioned medium to BGC-823 cells was lower compared with the 5-FU group (Figure 2e).

### 3.2. Conditioned Medium Promotes the Apoptosis of BGC-823 Cells

**Figure 3:**
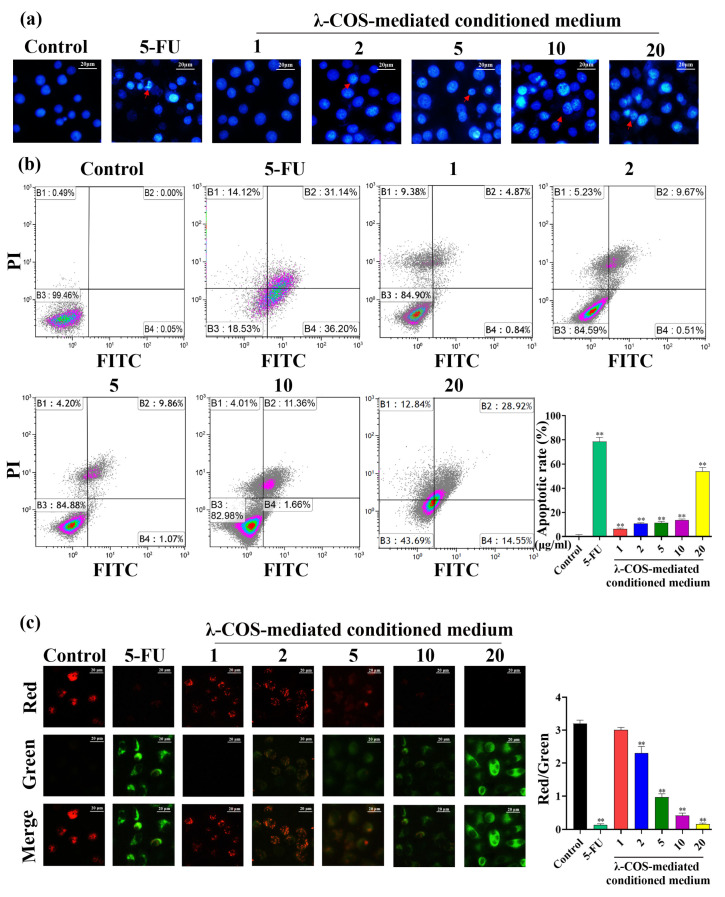
Conditioned medium promotes apoptotic progress in BGC-823 cells. (**a**) The effects of λ-COS-mediated conditioned medium and 5-FU on nuclear damage in BGC-823 cells. The cells were incubated with DAPI (10 μg/mL), and the images of the nuclei of BGC-823 cells were captured by a fluorescence microscope. The apoptotic bodies and swelling of the nucleus were pointed by red arrows. Bar = 20 μm. (**b**) The effects of conditioned medium and 5-FU on cell apoptotic rates in BGC-823 cells. The cells were incubated with Annexin V-FITC, followed by the detection performed by a flow cytometer. The experiment was repeated three times, and the representative pictures were selected for display. The excitation wavelengths of FITC and PI were both 488 nm, while the emission wavelengths of FITC and PI were 530 nm and 630 nm, respectively. (**c**) The effects of conditioned medium and 5-FU on the mitochondrial membrane in BGC-823 cells. The cells were exposed to a JC-1 staining solution and observed by the fluorescence microscope. Bar = 20 μm. ** *p* < 0.01 versus the control group.

Nuclear changes in treated BGC-823 cells were observed via DAPI staining. The control group cells were stained uniformly, revealing normal morphology, and the chromatin was homogeneously distributed in the nucleolus (Figure 3a). On the contrary, the intensity of blue fluorescence radiated from the treated BGC-823 cells decreased as the concentration of λ-COS used to generate the conditioned medium was increased. Furthermore, after 24 h of exposure to the conditioned medium, the cells displayed typical apoptotic characteristics, such as nuclear chromatin condensation and nuclear fragmentation. In addition, apoptotic bodies could be observed in the conditioned medium (20 μg/mL) and 5-FU-treated BGC-823 cells (Figure 3a).

The proportion of apoptotic BGC-823 cells was evaluated employing Annexin V-FITC/PI. After the BGC-823 cells were challenged by 10 μg/mL 5-FU, the apoptosis rate reached 78.43% (Figure 3b). When the BGC-823 cells were incubated with the conditioned medium, the apoptosis rate of cells was remarkably elevated in a concentration-dependent manner (*p* < 0.01, Figure 3b). Specifically, treatment with a conditioned medium (20 μg/mL) resulted in a peak apoptosis rate of 53.92%, suggesting that both the conditioned medium (20 μg/mL) and 5-FU had a significant stimulative effect on apoptosis in BGC-823 cells.

The transforming fluorescence of JC-1 from red to green can essentially reveal the extent of mitochondrial injury. An enhanced green fluorescence (JC-1 monomer) represents the depolarization of the mitochondrial membrane, while the enhanced red fluorescence (JC-1 polymer) indicates the polarization of the mitochondrial membrane. Therefore, we evaluated the effect of the conditioned medium on the mitochondrial membrane in BGC-823 cells via JC-1 staining. The cells in the control group just emitted red fluorescence (JC-1 polymer), while only green fluorescence (JC-1 monomer) was observed from the 5-FU group. Compared to the control group, an increase in the concentration of λ-COS in the conditioned medium led to a decrease in red fluorescence emission and an increase in green fluorescence detection (Figure 3c). These results suggested that, similar to 5-FU, the conditioned medium had the potential to induce mitochondrial injury. These results further demonstrated that the λ-COS-mediated conditioned medium could induce apoptosis in BGC-823 cells in a dose-dependent manner. 

### 3.3. λ-COS Activate Macrophages and Mediate Apoptosis by the Par-4 Signaling Pathway

**Figure 4:**
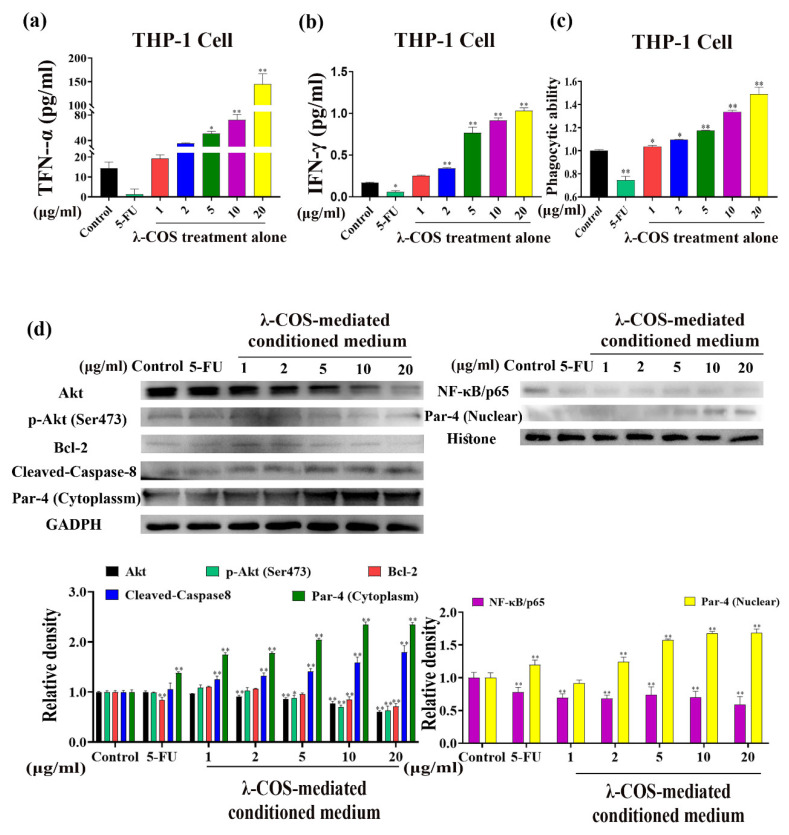
λ-COS activates macrophages, and the conditioned medium activates the Par-4 signaling pathway. (**a**,**b**) The effect of λ-COS on the secretions of TNF-α and IFN-γ in THP-1 cells. (**c**) The effect of λ-COS on the phagocytic ability of THP-1 cells. (**d**) Conditioned medium activated Par-4 signaling in BGC-823 cells. The conditioned medium increased the expression of Par-4 and the accumulation of nuclear Par-4, downregulating the expression of NF-κB. Conditioned medium also changed the expression of proteins associated with Par-4 signaling, which included B-cell lymphoma-2 (Bcl-2), Ak mouse strain thymoma (Akt), phosphor-Akt (p-Akt, Ser473), and Cleaved-Caspase-8. * *p* < 0.05 and ** *p* < 0.01 versus the control group.

In the conditioned medium culturing THP-1-derived macrophages, the levels of TNF-α and IFN-γ, compared with the control group, were markedly upregulated in a concentration-dependent manner after 24 h of incubation with λ-COS (*p* < 0.01, Figure 4a,b). In particular, 20 μg/mL λ-COS increased the levels of TNF-α and IFN-γ from 14.25 pg/mL to 144.90 pg/mL and from 0.17 pg/mL to 1.03 pg/mL, respectively. In contrast, the 5-FU downregulated the secreted cytokines. In addition, the phagocytic ability of THP-1-derived macrophages was also significantly enhanced, while 5-FU largely damaged it (*p* < 0.01, Figure 4c).

Studies have proven that the simultaneous action of TNF-α and IFN-γ can activate the Par-4 signaling pathway [22]. To further verify the mechanism of conditioned medium-induced apoptosis in BGC-823 cells, the expressions of Par-4 and other related proteins were assessed by Western blotting analysis. The results demonstrated that the conditioned medium significantly increased the level of nuclear Par-4, promoting its translocation from cytoplasm to the nucleus in a concentration-dependent manner, which was also accompanied by a reduction of NF-κB/p65 in the nucleus (*p* < 0.01). Along with the descended expression of p-Akt (Ser473), the functional regulator of Par-4, Akt, was significantly inhibited at the protein level (*p* < 0.01). The surging level of nuclear Par-4 also led to the reduction of the anti-apoptotic protein, Bcl-2, at the protein level, contributing to increased expression of cleaved-caspase-8 (Figure 4c). Compared with the control group, the conditioned medium (20 μg/mL) increased the expressions of nuclear Par-4 and cleaved-caspase-8 by 1.69-fold and 2.14-fold, respectively. At the same time, the expressions of NF-κB/p65, p-Akt, and Bcl-2 were decreased by 40.85%, 37.10%, and 28.83% compared with the control group, respectively.

### 3.4. λ-COS Inhibit Tumor Growth in Mice

**Figure 5:**
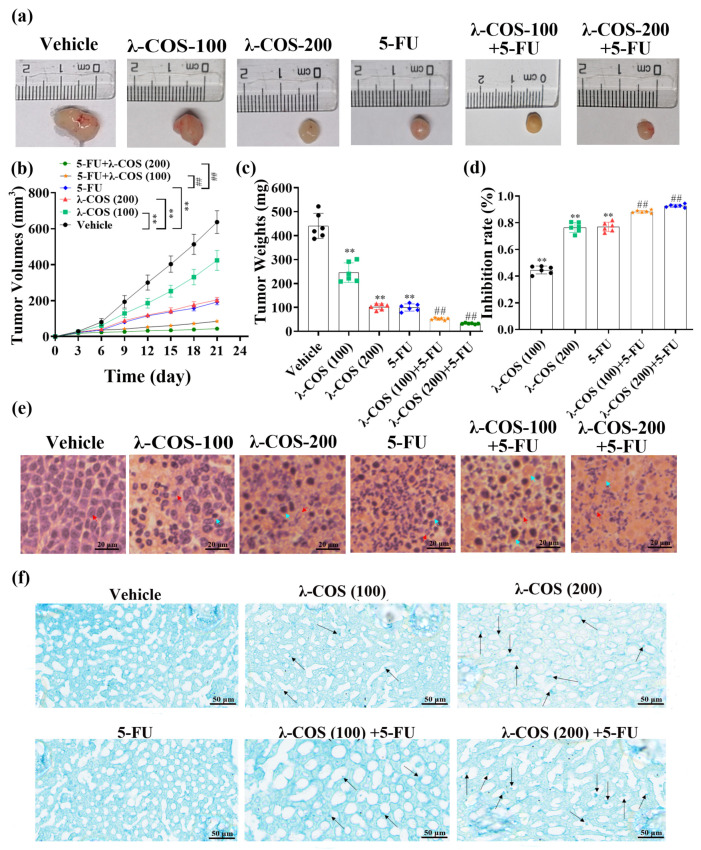
λ-COS and 5-FU together inhibit the growth of BGC-823 cells in vivo. (**a**) The macro-photo of the tumor after intraperitoneal injection for 21 d. (**b**) The variations in tumor volume during the 21 d of treatment in all groups. λ-COS were injected every day since the d 1st. The volume of the tumor was measured every 3 d. (**c**) The weight of tumor excised from BALB/c mice. (**d**) The effect of λ-COS on the tumor inhibition rate. The tumor inhibition rate was calculated with the formula (1 − average tumor weight in treated groups/average tumor weight in the vehicle group) × 100%. ** *p* < 0.01 versus the vehicle group. ^##^ *p* < 0.01 versus the 5-FU group. (**e**) The impact of λ-COS on the microstructure of tumor tissues. The tissues were stained by H&E. Bar = 20 μm. The normal tumor cells and their nuclear fragmentations are indicated by red arrows. The inflammatory cells are indicated by blue arrows. (**f**) Distribution of λ-COS in liver tissues by Alcian blue staining (indicated by black arrows).

To investigate the antitumor activity of λ-COS in vivo, we assessed the anti-gastric carcinoma effect of λ-COS in BALB/c mice by evaluating the activities of macrophages, the organs related to immunity, and the concentrations of cytokines in the serum after the tumor model was successfully established by subcutaneous injection of BGC-823 cells. The decreasing volume of tumors shows that λ-COS inhibited tumor growth over time in mice (Figure 5a).

Compared with the vehicle group, λ-COS administration for 21 d significantly reduced the mean volume and weight of tumors in a dose-dependent manner and surged inhibition rates (*p* < 0.01, Figure 5a–d). The tumor inhibition rates of λ-COS at 100 and 200 mg/kg were 44.6 ± 2.6% and 76.5 ± 3.4%, respectively. The tumor inhibition rate of the 5-FU alone group was 76.9 ± 3.2%. The combined group exhibited a higher tumor inhibitory capacity, which was increased by 13.1% and 21.2%, respectively, for 5-FU+λ-COS-100 and 5-FU+λ-COS-200 groups compared with the 5-FU group (*p* < 0.01, Figure 5a–d). These results demonstrated that λ-COS could enhance the antitumor activity of 5-FU in a concentration-dependent manner.

A histological assay was performed to examine tumor tissues and further evaluate the antitumor effect of λ-COS (Figure 5e). An intact architecture was observed from tumor tissues in the vehicle group, showing closely arranged tumor cells with large nuclei. λ-COS administration extensively disrupted tumor tissues. Moreover, λ-COS dose-dependently extended the necrotic areas of tumors. In the high-concentration λ-COS treatment group, 5-FU group, and combined group, the areas of infiltrating inflammatory cells and fragmented nuclear chromatin were noticeably expanded.

The presence of λ-COS in liver tissue was detected by a modified Alcian method [21], revealing the appearance of blue-staining heterogenous particles in the histiocytes and Kupffer-like cells that increased in number with λ-COS treatment concentration. Sparse and irregular inclusion bodies were observed in the Kupffer-like liver cells, suggesting that λ-COS might penetrate the intestinal barrier to reach liver tissues (Figure 5f).

### 3.5. λ-COS Enhances Immune Function and Alleviates 5-FU-Induced Immunosuppression in Mice

**Figure 6:**
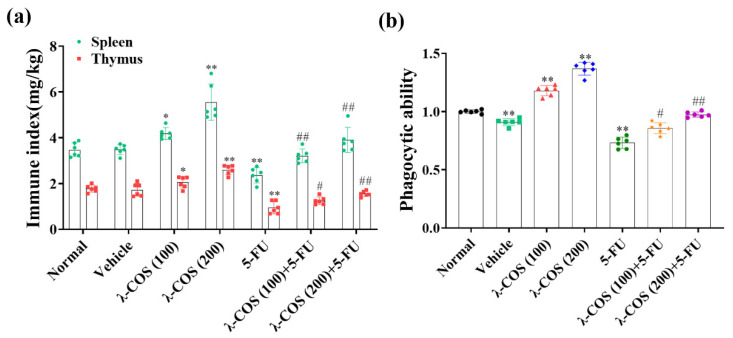
The immune response of BALB/c mice is promoted by λ-COS. (**a**) The effect of λ-COS on the indexes of spleen and thymus in mice. The indexes were calculated by comparing their weights with the total weights of mice. (**b**) The phagocytic ability of peritoneal macrophages after administration with λ-COS in BALB/c mice. The cells were incubated with neutral red to indicate their phagocytic ability (*n* = 6). * *p* < 0.05 and ** *p* < 0.01 versus the normal group. ^#^ *p* < 0.05 and ^##^ *p* < 0.01 versus the 5-FU group.

Since the spleen and thymus are two of the primary immune organs, their indexes were used to estimate the effect of λ-COS on immune functions. When the mice were injected with 5-FU alone, in comparison with the normal group, the indexes of both the spleen and thymus were significantly decreased by 31.9 ± 9.4% and 47.3 ± 11.0%, respectively. Compared with the normal group, λ-COS, on the contrary, exhibited antitumor activity while increasing the indexes of the organs in a dose-dependent manner. High-dose treatment of λ-COS increased the indexes of the spleen and thymus by 1.60 ± 0.18-fold and 1.47 ± 0.18-fold, respectively. When 5-FU and λ-COS were combined, the indexes of the spleen and thymus were increased by 1.69 ± 0.33-fold and 1.73 ± 0.38-fold compared with the 5-FU alone group, respectively (Figure 6a). These results revealed that λ-COS could enhance the activity of the spleen and thymus in BALB/C mice, alleviating the immunosuppressive effect from 5-FU.

We next examined the impacts of λ-COS on the phagocytic capacity of macrophages in mice. The phagocytic capacity of macrophages was significantly lower in the vehicle group compared with the normal group (*p* < 0.01), hinting that tumor invasion weakened the immunity of BALB/c mice. λ-COS (200 mg/kg) considerably enhanced the phagocytic ability of macrophages in comparison with the normal group (*p* < 0.01). Compared with the normal group, the activities of peritoneal macrophages in the 5-FU group were significantly downregulated (*p* < 0.01), while its phagocytic ability was efficiently improved with the λ-COS supplement (Figure 6b). These results suggested that λ-COS exposure elicited immune cell responses in BGC-823-challenged BALB/C mice.

### 3.6. λ-COS Increase Cytokine Expression and Promote Pro-Apoptotic Proteins Levels in Tumor Tissues

**Figure 7:**
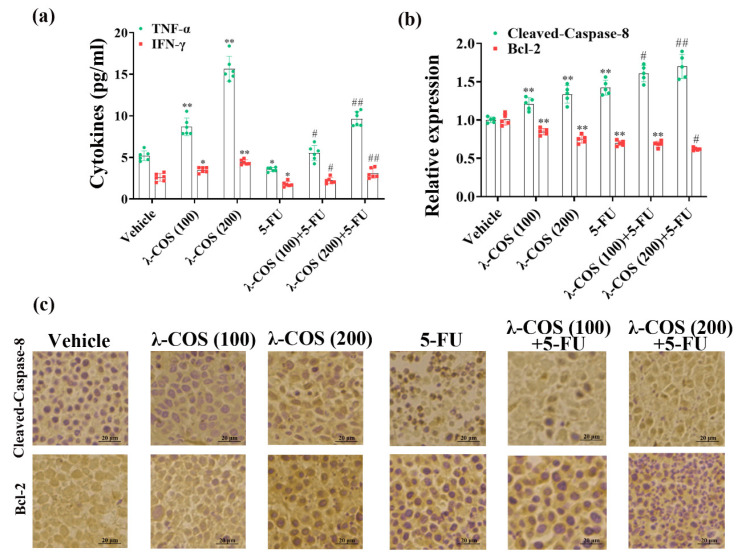
The expressions of cytokines and apoptosis-related proteins are enhanced by λ-COS. (**a**) The effect of λ-COS on the concentrations of TNF-α and IFN-γ in mouse serum. The blood samples were collected from the eyeballs of mice. The levels of cytokines in the serum samples were detected by ELISA. (**b**) The expressions of cleaved-caspase-8 and Bcl-2 in tumor tissues of all groups. The expressions of the apoptosis-related proteins were evaluated via immunohistochemical detection. * *p* < 0.05 and ** *p* < 0.01 versus the vehicle group. ^#^ *p* < 0.05 and ^##^ *p* < 0.01 versus the 5-FU group. (**c**) The mean densities of cleaved-caspase-8 and Bcl-2 in tumor tissues of all groups were evaluated by using ImageJ software. Bar = 20 μm.

The impacts of λ-COS on the secretions of TNF-α and IFN-γ were assessed to obtain more solid evidence of the antineoplastic mechanism in mice. Our results demonstrated that the levels of TNF-α and IFN-γ in λ-COS-treated groups were remarkably higher than the vehicle group (*p* < 0.01). Especially, the concentrations of TNF-α and IFN-γ reached 15.65 pg/mL and 4.42 pg/mL in the 200 mg/kg λ-COS group, which were 3.13 ± 0.52-fold and 1.76 ± 0.38-fold higher than the levels in the vehicle group, respectively. The levels of TNF-α and IFN-γ were remarkably decreased in the 5-FU group compared with the vehicle group (*p* < 0.05, Figure 7a). The combination of λ-COS and 5-FU could restore the TNF-α and IFN-γ that were impaired after 5-FU exposure, showing that λ-COS had an ameliorative effect on immune activity in mice. Besides, the data demonstrated that the antineoplastic effect of λ-COS in BGC-823-challenged mice might be related to their capacity to upregulate TNF-α and IFN-γ.

To further explore how λ-COS induced apoptosis in tumor tissue, the expressions of cleaved-caspase-8 and Bcl-2 in the tumor samples were analyzed by immunohistochemical assay. The results of immunohistochemical detection reveal that λ-COS, in a dose-dependent manner, enhanced the expression of pro-apoptotic cleaved-caspase-8 protein, along with the downregulation of Bcl-2 protein in solid tumors. Remarkably, 20 mg/kg 5-FU and 200 mg/kg λ-COS significantly upregulated cleaved-caspase-8 at the protein level compared with the 5-FU group (*p* < 0.01, Figure 7b,c). 

## 4. Discussion

λ-CGN is a polysaccharide extracted from red algae. Its oligosaccharide has been confirmed to possess immunomodulatory, antitumor, and antiviral properties due to its smaller molecular weight, better water solubility, and more fully exposed active groups [23]. However, the structural characteristics and concomitant bioactivities of COS are significantly different, which can be attributed to their diverse sources or isolation methods. In the present study, λ-COS was obtained from the food-grade λ-CGN by acid degradation, gradient ethanol precipitation, column chromatography separation, and ultrafiltration. Our results demonstrated the high purity of λ-COS without protein and nucleic acid.

In our previous study, we found that degraded λ-CGN fails to induce the apoptosis of colon cancer cells. However, a low concentration of CGN can induce cell apoptosis and reduce the transepithelial electrical resistance of the Caco-2 cell monolayers in the co-culture model of Caco-2 and THP-1 cells via the TLR4 and ERK1/2-mediated upregulation of TNF-α [17,18]. These results indicate that degraded λ-CGN-mediated TNF-α release may mainly contribute to cellular injury in Caco-2 monolayers. In the present study, we assessed the anti-gastric carcinoma activity of λ-COS in vitro and in vivo using 2D cell culture and animal models. CCK-8 assay indicated that λ-COS would have no significant impact on BGC-823 cells. In contrast, the growth of BGC-823 cells was inhibited by the λ-COS-mediated conditioned medium of macrophages in a dose-dependent manner, and it promoted the apoptosis of BGC-823 cells. A higher PI signal indicated more late apoptotic and necrotic cells, while the FITC+PI- cell population represented early apoptotic cells. With the increase of λ-COS concentration in the conditioned medium, we found that the λ-COS-mediated conditioned medium could significantly induce apoptosis in BGC-823 cells.

Carrageenan is degraded by gastric acid in the gastrointestinal tract to produce oligo-carrageenan [24]. Therefore, mice were given λ-COS by intraperitoneal injection in this study. Some research has indicated that CGN may pass across the epithelium of the small intestine, colon, or caecum and accumulate in the lamina propria, where it would then be ingested by mast cells, finally passing via the blood to tissues [21,25]. In our present study, we employed a modified Alcian blue staining method to detect λ-COS in the liver. We observed the presence of positive hetero-staining particles in tissues, and the quantity was increased with the increased concentration of λ-COS. This finding strongly suggested that λ-COS might be present in the tissues. However, we could not exclude the possible existence of sulfated glycosaminoglycans. This possibility remains to be further studied. As a hinge of the immune response, macrophages can devour pathogens and emit various cytokines [26]. Therefore, we further investigated whether λ-COS affected the secretory ability of THP-1-derived macrophages and the phagocytic activity of macrophages in BALB/c mice, aiming to verify the immunomodulatory property of λ-COS. Maeβ et al. have demonstrated that CGN can enhance the phagocytic capability of RAW 264.7 cells while advancing cell proliferation [27]. Consistent with this result, we also found that λ-COS markedly improved the phagocytic ability of macrophages, which could be considered a signal of immunoenhancement. Meanwhile, the simultaneously increased TNF-α and IFN-γ levels were also observed after λ-COS treatment in the medium incubated with THP-1-derived macrophages. Our previous research has confirmed that κ-CGN can strengthen the secretion of TNF-α in the co-culture system, which is a key participant in Caco-2 apoptosis [17,18]. However, our λ-COS did not induce apoptosis in THP-1 cells in this study but rather stimulated the secretion of TNF-α. This could be due to the fact that λ-COS at 20 μg/mL just increased the level of TNF-α from 14.25 pg/mL to 144.90 pg/mL in THP-1 cells, as compared to the control. In comparison, κ-CGN treatment at 10 μg/mL resulted in the secretion of 5772.2 pg/mL of TNF-α in the medium of Caco-2 cells. Therefore, κ-CGN could induce the disruption of intestinal epithelial Caco-2 monolayers in the co-culture cell model of Caco-2 and THP-1 cells by activating death receptors like Fas, which can mediate the exogenous pathway of apoptosis in Caco-2 cells [17]. Interestingly, MCF-7 cells are known to be resistant to soluble TNF-α due to their expression of the TNF-α leader sequence [28]. TNF-α can also induce FoxMI expression to resist cell apoptosis in hepatocellular carcinoma [29]. This could potentially explain why some cancer cell lines are not sensitive to λ-COS-induced conditioned medium. However, IFN-γ, the cytokine that was long restricted to NK cells and T cells, can also be induced by λ-COS via stimulating THP-1-derived macrophages and thus inhibit gastric carcinoma [30]. There stands a good chance that the combination of these cytokines, not TNF-α alone, is the vital contributor to the apoptotic progress of BGC-823 cells via λ-COS treatment. Besides, PMA can only induce THP-1 cells to differentiate into M0 macrophages. In order to be further polarized to M1 macrophages, the tumor-resisting macrophages that can secret TNF-α, THP-1 cells are always treated with IFN-γ or LPS after incubating with PMA [31]. Hence, the transforming of THP-1 cells-derived M0 macrophages to M1 macrophages is probably due to the promotion of IFN-γ self-secretion in THP-1 cells, which are both mediated by λ-COS. Since M1 macrophages are reported to be capable of targeting tumor cells, their activation would be an efficient way to kill tumor cells, which can be realized by the administration of λ-COS [32]. In addition, we also found that the indexes of the thymus and spleen could be enhanced through λ-COS treatment in gastric adenocarcinoma cells BGC-823 xenografted mice, which was consistent with the results in H22 hepatoma-bearing mice [33]. Given the immunomodulatory property of λ-COS, it is highly suggested that antitumor immunity occupies a crucial place in the antitumor mechanism of λ-COS.

Par-4, a nature tumor suppressor protein, plays a significant role in inducing tumors to undergo apoptosis, which has been deemed a target for cancer treatment [34,35]. The overexpression of Par-4 is insufficient for apoptosis induced via Par-4 signaling, indicating that the trafficking of Par-4 from the cytoplasm to the nuclear is indispensable [36]. If tumor cells remain Par-4 in the cytoplasm, the cells would be resistant to Par-4-enticed apoptosis [37]. Besides, there is a report pointing out that the low level of Par-4 is related to the growth of gastric carcinoma, making it more aggressive [38]. In our present research, we found that the conditioned medium not only surged the level of Par-4 in the cytoplasm but also enhanced its accumulation in the nucleus of BGC-823 cells in a λ-COS-dependent manner. Interestingly, according to earlier research, the treatment of TNF-α or IFN-γ alone does not exhibit a significant influence on the level of Par-4, while their combination can significantly upregulate nuclear Par-4 [22]. Therefore, it is reasonable to deem that Par-4 signaling can be activated by co-stimulation of TNF-α and IFN-γ, which may be why BGC-823 cells become sensitive to λ-COS. 

Meanwhile, Par-4 can also be negatively regulated by Akt. The constant stimulation of Akt would lead cancer cells to resist various types of chemotherapy [39]. Goswami et al. have demonstrated that Akt can bind to Par-4, phosphorylating it to inhibit its nuclear accumulation [40]. In the present research, we found that the agent significantly suppressed the expression of total and phosphorylated (Ser473) Akt, deactivating the PI3k-Akt pathway. Furthermore, it has been reported that Par-4 in the cytoplasm can interact with Fbxo4, which mediates its ubiquitylation and proteasomal degradation [41]. An earlier study has shown that the injection of spinal TNF-α can decrease the expression of Fbxo4 in mice [42]. Thus, TNF-α might also be able to suppress the degradation of cytoplasm Par-4 by suppressing Fbxo4 in BGC-823 cells. This could potentially explain the re-regulation of Par-4 levels in both the cytoplasm and nucleus of cancer cells exposed to λ-COS. However, further experimental verification is required to confirm this hypothesis. The suppressions of NF-κB and Bcl-2 are involved in the mechanisms of Par-4-induced apoptosis [43]. A recent report has revealed that CGN can inhibit the nuclear accumulation of NF-κB/p65 in N9 cells [44]. In the present study, we found that λ-COS-mediated conditioned medium downregulated the expressions of NF-κB and Bcl-2. Therefore, we speculated that in addition to the effects of TNF-α or IFN-γ; the leftover λ-COS in the conditioned medium might also have a synergistic effect on blocking the NF-κB/Bcl-2 pathway. Bcl-2 is known for enhancing the resistance of MIA-PaCa-2 pancreatic cancer cells to chemical agents [45]. Therefore, one of the antitumor mechanisms that λ-COS-induced cytokines possess may rely on the downregulation of Bcl-2, making cancer cells more vulnerable to the enhanced immune system and chemotherapy.

Another apoptotic mechanism induced via Par-4 signaling is attributed to the trafficking of Fas or FasL, which would activate caspase-8, a crucial mediator of apoptosis [46]. Moreover, the activity of caspase-8 is also a prerequisite for stimulating Par-4 signaling to induce cell apoptosis [47]. Interestingly, neither the inhibition of NF-κB activity nor the sensitization of the Fas pathway can induce cell apoptosis alone [48]. However, in vitro and in vivo data showed that the λ-COS-induced cytokines could downregulate the expression of Akt, stimulating the Par-4 signaling to make both events parallel. While λ-COS has shown potential in enhancing the immune system and inducing apoptosis of gastric carcinoma cells through Par-4 signaling, it should be noted that λ-COS also increased the secretion of TNF-α, a proinflammatory cytokine, which may have negative effects that require further evaluation. In a study by Yin et al., it has been found that both λ-COS- and κ-COS-degrading bacteria could induce intestinal inflammation in germ-free mice [49]. These findings highlight the importance of considering the safety and potential adverse effects of λ-COS in future applications as an antitumor therapy. Further research and experimental verification are needed to better understand the implications of TNF-α secretion and the overall safety profile of λ-COS.

5-FU, as a first-line antineoplastic agent, is widely used for the treatment of gastric carcinoma, and has been used in clinical treatments for years [50]. However, 5-FU could lead to several side effects including drug resistance as well as immunosuppression, which were widely proven [51]. In this study, we proved that combination of λ-COS at the dose of 100 and 200 mg/kg with 5-FU had a synergistic effect for enhancing antitumor activity on BGC-823-cells-induced heterogeneity mice, compared with that of 5-FU alone.

## 5. Conclusions

Taken together, this study revealed the anti-gastric carcinoma activity of λ-COS from the aspect of immunomodulatory mechanism in vitro and in vivo. Although λ-COS had no cytotoxicity toward BGC-823 cells, λ-COS could activate macrophages and thus suppress the growth of BGC-823 cells by triggering cell apoptosis. λ-COS-exposed macrophages primarily enhanced gastric carcinoma cells’ apoptosis by activating Par-4 signaling. Moreover, the in vivo anticancer activity of λ-COS might be realized by restoring organs related to immunity, regulating cellular immune responses, and enhancing TNF-α and IFN-γ secretions. Importantly, λ-COS, in combination with 5-FU, might ameliorate the anti-gastric carcinoma impact and reduce the side effects of 5-FU on the immune system. On the whole, λ-COS, a potential antitumor agent, would bid fair to be an appropriate immune adjuvant in the chemotherapy.

## Data Availability

All data needed to evaluate the conclusions in the paper are present in the paper.

## Supplementary Materials

The following supporting information can be downloaded at: https://www.mdpi.com/article/10.3390/nu15092044/s1, Table S1. Ions identified for the oligo-λ-carrageenans in the positive ion-mode ESIMS. Figure S1. ESIMS spectrum of disaccharide (λ-2) with positive model. Figure S2. ESIMS spectrum of heptasaccharide (λ-7) with positive model. Figure S3. ESIMS spectrum of tridesaccharide (λ-13) with positive model. Figure S4. The effects of λ-COS and λ-COS-mediated conditioned medium on the growth of multiple cancer cell lines. Including PC-9, MCF-7, BV-2, HT-29, Caco-2 and HepG2 cell lines, the survival rates of cells cultured with different dosages of λ-COS and λ-COS-mediated conditioned medium were evaluated by CCK-8 assay (*n* = 3).
