# Supplementary material for: The Antitumor Potential of λ-Carrageenan Oligosaccharides on Gastric Carcinoma by Immunomodulation
Source: Nutrients. 2023 Apr 24;15(9):2044. doi: 10.3390/nu15092044 (PMC10181092; doi:10.3390/nu15092044)
Supplement: Supplementary file 1 [file nutrients-15-02044-s001.zip › nutrients-2298192-supplementary.pdf]

The Anti-Tumor Potential of λ-Carrageenan Oligosaccharides on Gastric Carcinoma by Immunomodulation

Min Tang, Leilei Zhai, Juanjuan Chen, Feng Wang, Haimin Chen, Wei Wu

Supplementary Tables

Supplementary Table S1. Ions identified for the oligo-λ-carrageenans in the positive ion-mode ESIMS

Oligosaccharide (A-G4S)n	Positive ion-mode	m/z
Disaccharide	$[(D2S6S\text{-}G2S)\text{-}3SO_3\text{+}Na\text{-}2H_2O]^+$	329
λ-2	$[(D2S6S\text{-}G2S)\text{-}2SO_3\text{+}(C_7H_{15}NH_3)\text{+}Na\text{-}H_2O]^+$	542
	$[(D2S6S\text{-}G2S)\text{-}SO_3\text{+}(C_7H_{15}NH_3)_2\text{+}Na]^+$	755
	$[(D2S6S\text{-}G2S)\text{+}(C_7H_{15}NH_3)_3\text{+}Na\text{+}H_2O]^+$	968
	$[(D2S6S\text{-}G2S)\text{+}SO_3\text{+}(C_7H_{15}NH_3)_4\text{+}Na\text{+}H_2O]^+$	1181
Trisaccharide	$[(D2S6S\text{-}G2S)D2S6S\text{+}(C_7H_{15}NH_3)_6\text{-}2H_2O]^+$	1560
λ-3	$[(D2S6S\text{-}G2S)D2S6S\text{+}SO_3\text{+}(C_7H_{15}NH_3)_5\text{+}2Na\text{+}H_2O]^+$	1639
Tetrasaccharide	$[(D2S6S\text{-}G2S)_2\text{-}4SO_3\text{+}(C_7H_{15}NH_3)_4\text{+}H_2O]^{2+}$	653
λ-4	$[(D2S6S\text{-}G2S)_2\text{-}2SO_3\text{+}(C_7H_{15}NH_3)_6\text{+}H_2O]^{2+}$	848
	$[(D2S6S\text{-}G2S)_2\text{+}(C_7H_{15}NH_3)_8\text{+}H_2O]^{2+}$	1043
Pentasaccharide	$[(D2S6S\text{-}G2S)_2D2S6S\text{-}3SO_3\text{+}(C_7H_{15}NH_3)_6\text{+}3Na\text{-}H_2O]^{2+}$	983
λ-5	$[(D2S6S\text{-}G2S)_2D2S6S\text{-}SO_3\text{+}(C_7H_{15}NH_3)_7\text{+}2Na\text{+}H_2O]^{2+}$	1128
	$[(D2S6S\text{-}G2S)_2D2S6S\text{-}3SO_3\text{+}(C_7H_{15}NH_3)_3\text{+}3Na\text{+}H_2O]^+$	1656
	$[(D2S6S\text{-}G2S)_2D2S6S\text{-}2SO_3\text{+}(C_7H_{15}NH_3)_4\text{+}3Na\text{+}H_2O]^+$	1851
Hexasaccharide	$[(D2S6S\text{-}G2S)_3\text{-}2SO_3\text{+}(C_7H_{15}NH_3)_6\text{+}3Na\text{-}2H_2O]^{2+}$	1137
λ-6	$[(D2S6S\text{-}G2S)_3\text{-}SO_3\text{+}(C_7H_{15}NH_3)_7\text{+}3Na\text{-}2H_2O]^{2+}$	1234
	$[(D2S6S\text{-}G2S)_3\text{+}(C_7H_{15}NH_3)_9\text{+}2Na\text{-}2H_2O]^{2+}$	1379
	$[(D2S6S\text{-}G2S)_3\text{-}SO_3\text{+}(C_7H_{15}NH_3)_{10}\text{+}H_2O]^{2+}$	1400
Heptasaccharide	$[(D2S6S\text{-}G2S)_3D2S6S\text{-}6SO_3\text{+}(C_7H_{15}NH_3)_3\text{+}4Na\text{-}H_2O]^{2+}$	984
λ-7	$[(D2S6S\text{-}G2S)_3D2S6S\text{-}5SO_3\text{+}(C_7H_{15}NH_3)_4\text{+}4Na\text{-}H_2O]^{2+}$	1081
	$[(D2S6S\text{-}G2S)_3D2S6S\text{-}4SO_3\text{+}(C_7H_{15}NH_3)_6\text{+}3Na]^{2+}$	1234
	$[(D2S6S\text{-}G2S)_3D2S6S\text{+}(C_7H_{15}NH_3)_{11}\text{+}2Na\text{-}2H_2O]^{2+}$	1656
	$[(D2S6S\text{-}G2S)_3D2S6S\text{-}6SO_3\text{+}(C_7H_{15}NH_3)_3\text{+}3Na\text{-}6H_2O]^+$	1851
	$[(D2S6S\text{-}G2S)_3D2S6S\text{-}5SO_3\text{+}(C_7H_{15}NH_3)_4\text{+}3Na\text{-}6H_2O]^+$	2047
Octasaccharide	$[(D2S6S\text{-}G2S)_4\text{-}4SO_3\text{+}(C_7H_{15}NH_3)_7\text{+}4Na\text{-}2H_2O]^{3+}$	937
λ-8	$[(D2S6S\text{-}G2S)_4\text{-}6SO_3\text{+}(C_7H_{15}NH_3)_6\text{+}2Na\text{-}3H_2O]^{2+}$	1235
	$[(D2S6S\text{-}G2S)_4\text{-}5SO_3\text{+}(C_7H_{15}NH_3)_6\text{+}3Na\text{+}2H_2O]^{2+}$	1332

	[(D2S6S-G2S)$_4$-2SO$_3$+(C$_7$H$_{15}$NH$_3$)$_9$+3Na-2H$_2$O]$^{2+}$	1591
	[(D2S6S-G2S)$_4$-2SO$_3$+(C$_7$H$_{15}$NH$_3$)$_{12}$+ H$_2$O]$^{2+}$	1757
Nonasaccharide	[(D2S6S-G2S)$_4$D2S6S-11SO$_3$+(C$_7$H$_{15}$NH$_3$)$_3$+4Na-	518
λ-9	4H$_2$O]$^{4+}$	1090
	[(D2S6S-G2S)$_4$D2S6S-6SO$_3$+(C$_7$H$_{15}$NH$_3$)$_{10}$+Na-H$_2$O]$^{3+}$	1188
	[(D2S6S-G2S)$_4$D2S6S-4SO$_3$+(C$_7$H$_{15}$NH$_3$)$_{11}$+2Na-H$_2$O]$^{3+}$	1583
	[(D2S6S-G2S)$_4$D2S6S-5SO$_3$+(C$_7$H$_{15}$NH$_3$)$_8$+3Na-H$_2$O]$^{2+}$	
Decasaccharide	[(D2S6S-G2S)$_5$-7SO$_3$+(C$_7$H$_{15}$NH$_3$)$_{12}$+5H$_2$O]$^{4+}$	937
λ-10	[(D2S6S-G2S)$_5$-8SO$_3$+(C$_7$H$_{15}$NH$_3$)$_8$+2Na-3H$_2$O]$^{3+}$	1035
	[(D2S6S-G2S)$_5$-7SO$_3$+(C$_7$H$_{15}$NH$_3$)$_{10}$+5H$_2$O]$^{2+}$	1758
	[(D2S6S-G2S)$_5$-5SO$_3$+(C$_7$H$_{15}$NH$_3$)$_{12}$+5H$_2$O]$^{2+}$	1954
Hendesaccharide	[(D2S6S-G2S)$_5$D2S6S-4SO$_3$+(C$_7$H$_{15}$NH$_3$)$_{16}$-4H$_2$O]$^{3+}$	1536
λ-11	[(D2S6S-G2S)$_5$D2S6S-	1689
	9SO$_3$+(C$_7$H$_{15}$NH$_3$)$_7$+3Na+4H$_2$O]$^{2+}$	1787
	[(D2S6S-G2S)$_5$D2S6S-	2038
	8SO$_3$+(C$_7$H$_{15}$NH$_3$)$_8$+3Na+4H$_2$O]$^{2+}$	
	[(D2S6S-G2S)$_5$D2S6S-	
	6SO$_3$+(C$_7$H$_{15}$NH$_3$)$_{11}$+2Na+5H$_2$O]$^{2+}$	
Dodecasaccharid	[(D2S6S-G2S)$_6$-7SO$_3$+(C$_7$H$_{15}$NH$_3$)$_{15}$-2H$_2$O]$^{4+}$	1132
e	[(D2S6S-G2S)$_6$-9SO$_3$+(C$_7$H$_{15}$NH$_3$)$_9$+3Na+6H$_2$O]$^{3+}$	1296
λ-12	[(D2S6S-G2S)$_6$-8SO$_3$+(C$_7$H$_{15}$NH$_3$)$_{12}$+Na+H$_2$O]$^{3+}$	1393
	[(D2S6S-G2S)$_6$-8SO$_3$+(C$_7$H$_{15}$NH$_3$)$_8$+4Na]$^{2+}$	1884
	[(D2S6S-G2S)$_6$-7SO$_3$+(C$_7$H$_{15}$NH$_3$)$_{12}$+Na+3H$_2$O]$^{2+}$	2149
Tridesaccharide	[(D2S6S-G2S)$_6$D2S6S-	1197
λ-13	8SO$_3$+(C$_7$H$_{15}$NH$_3$)$_{14}$+2Na+3H$_2$O]$^{4+}$	1393
	[(D2S6S-G2S)$_6$D2S6S-10SO$_3$+(C$_7$H$_{15}$NH$_3$)$_{11}$+2Na-	1451
	3H$_2$O]$^{3+}$	1490
	[(D2S6S-G2S)$_6$D2S6S-9SO$_3$+(C$_7$H$_{15}$NH$_3$)$_{12}$+2Na-	
	4H$_2$O]$^{3+}$	
	[(D2S6S-G2S)$_6$D2S6S-10SO$_3$+(C$_7$H$_{15}$NH$_3$)$_{13}$+3H$_2$O]$^{3+}$	
Tetradecasacchar	[(D2S6S-G2S)$_7$-9SO$_3$+(C$_7$H$_{15}$NH$_3$)$_{13}$+2Na-H$_2$O]$^{3+}$	1587
ide	[(D2S6S-G2S)$_7$-9SO$_3$+(C$_7$H$_{15}$NH$_3$)$_{15}$+5H$_2$O]$^{2+}$	1686
λ-14		

Pentadesaccharide λ-15	$[(D2S6S\text{-}G2S)_7D2S6S\text{-}12SO_3+(C_7H_{15}NH_3)_{12}+6Na\text{-}2H_2O]^{7+}$	686

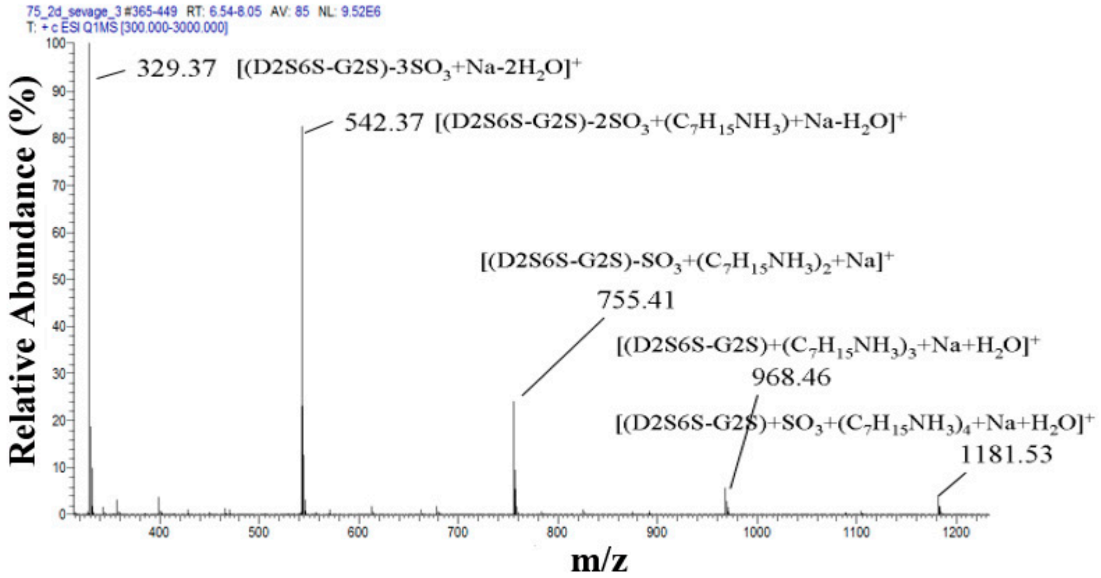

Supplementary Figure S1. ESIMS spectrum of disaccharide (λ-2) with positive model.

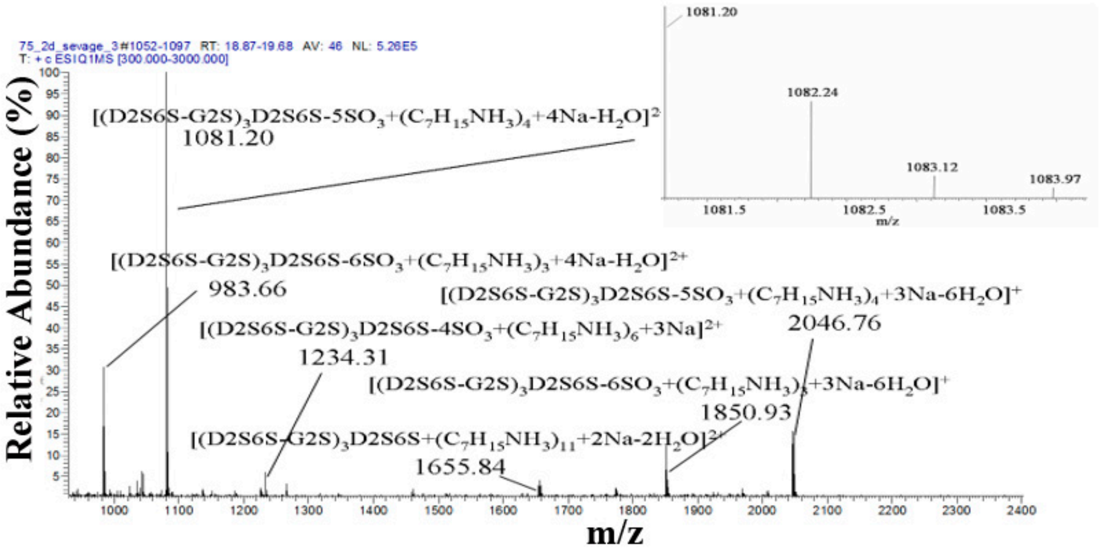

Supplementary Figure S2. ESIMS spectrum of heptasaccharide (λ-7) with positive model.

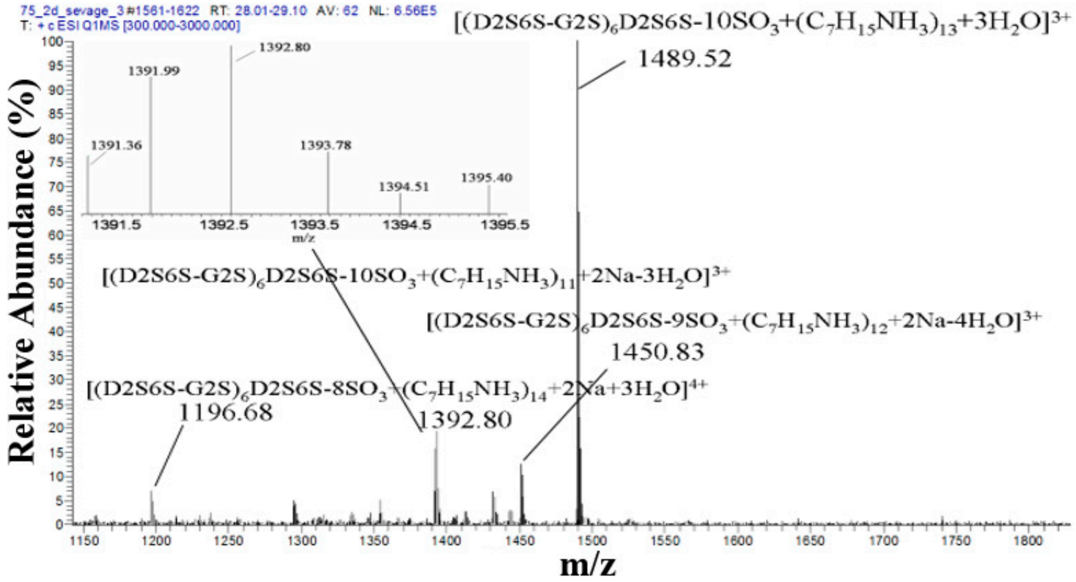

Supplementary Figure S3. ESIMS spectrum of tridesaccharide (λ-13) with positive model.

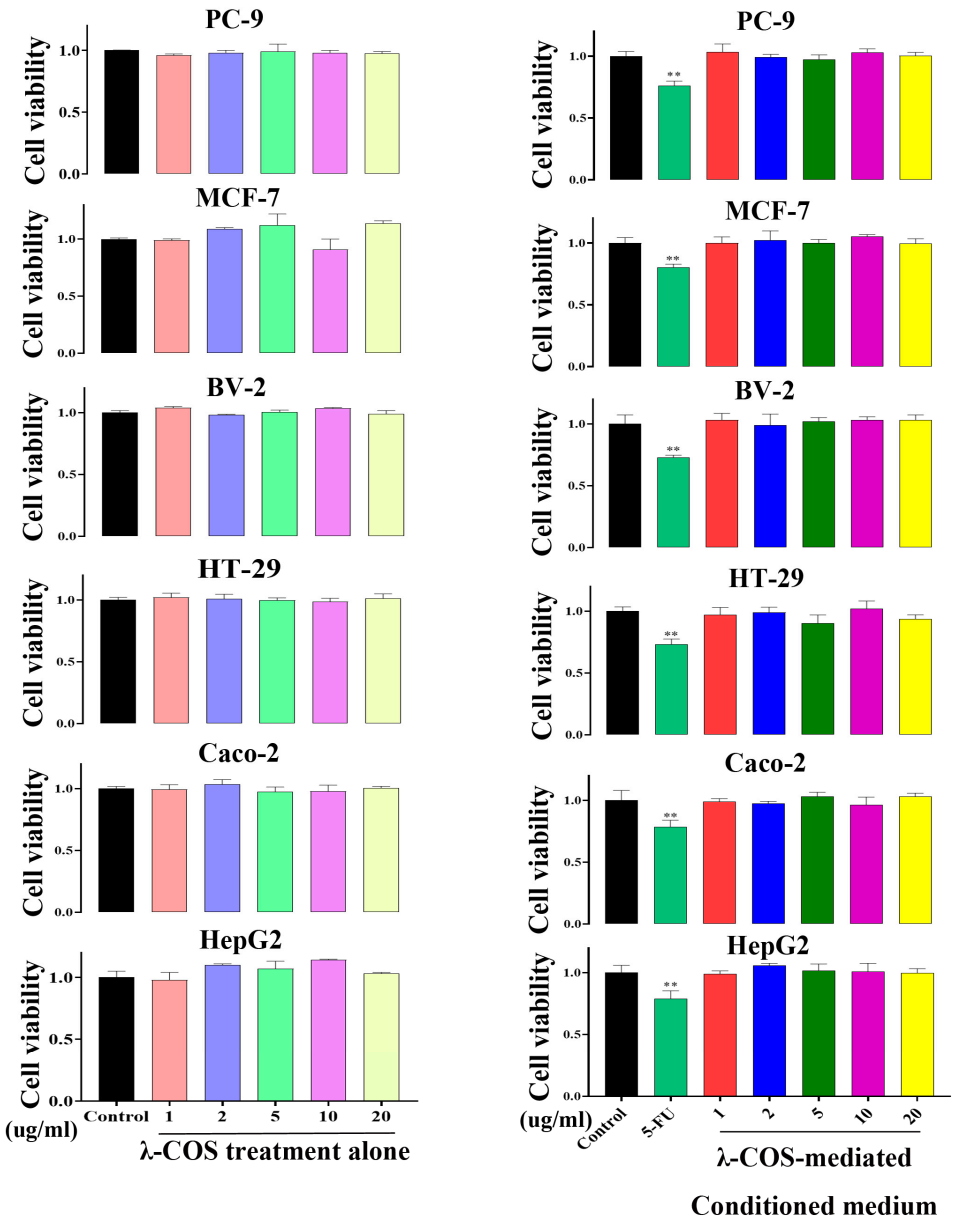

Supplementary Figure S4. The effects of λ-COS and λ-COS-mediated conditioned medium on the growth of multiple cancer cell lines. Including PC-9, MCF-7, BV-2, HT-29, Caco-2 and HepG2 cell lines, the survival rates of cells cultured with different dosages of λ-COS and λ-COS-mediated conditioned medium were evaluated by CCK-8 assay (*n* = 3).
